# Assessment of Ventral Tail Base Surface Temperature for the Early Detection of Japanese Black Calves with Fever

**DOI:** 10.3390/ani13030469

**Published:** 2023-01-29

**Authors:** Yosuke Sasaki, Yoshihiro Iki, Tomoaki Anan, Jun Hayashi, Mizuho Uematsu

**Affiliations:** 1 Department of Agriculture, School of Agriculture, Meiji University, Kawasaki 214-8571, Japan; 2 Center for Animal Disease Control, University of Miyazaki, Miyazaki 889-2192, Japan; 3 Miyazaki Agricultural Mutual Aid Association, Miyazaki 880-0852, Japan

**Keywords:** backgrounding operation, body surface temperature, bovine respiratory disease, Japanese Black, wearable sensor

## Abstract

**Simple Summary:**

Bovine respiratory disease is the most common and costly disease in beef cattle. We previously elucidated risk factors for bovine respiratory disease in Japanese Black calves reared in a backgrounding operation. In this operation, calves are reared in an intensive freestall system that contains several calves, and it is important to develop an early detection system to identify calves with any problems at an early stage. This study examined the assessment of the ventral tail base surface temperature (ST) for the early detection of Japanese Black calves with fever. A wearable wireless tail ST sensor was attached to the surface of the ventral tail base of each calf at its introduction to the backgrounding farm. Data obtained from the ST sensor were analyzed using supervised machine learning algorithms that use a random forest. This study found that the early detection of calves with fever can be predicted by monitoring the ventral tail base ST using a wearable wireless sensor. This knowledge could contribute to decreasing labor and the burden on clinical veterinarians.

**Abstract:**

The objective in the present study was to assess the ventral tail base surface temperature (ST) for the early detection of Japanese Black calves with fever. This study collected data from a backgrounding operation in Miyazaki, Japan, that included 153 calves aged 3–4 months. A wearable wireless ST sensor was attached to the surface of the ventral tail base of each calf at its introduction to the farm. The ventral tail base ST was measured every 10 min for one month. The present study conducted an experiment to detect calves with fever using the estimated residual ST (rST), calculated as the estimated rST minus the mean estimated rST for the same time on the previous 3 days, which was obtained using machine learning algorithms. Fever was defined as an increase of ≥1.0 °C for the estimated rST of a calf for 4 consecutive hours. The machine learning algorithm that applied was a random forest, and 15 features were included. The variable importance scores that represented the most important predictors for the detection of calves with fever were the minimum and maximum values during the last 3 h and the difference between the current value and 24- and 48-h minimum. For this prediction model, accuracy, precision, and sensitivity were 98.8%, 72.1%, and 88.1%, respectively. The present study indicated that the early detection of calves with fever can be predicted by monitoring the ventral tail base ST using a wearable wireless sensor.

## 1. Introduction

In the Japanese beef industry, the most common breed is the Japanese Black, also known as Wagyu. The Japanese Black has a superior ability to produce marbled beef compared with other beef breeds [1,2]. Production costs for rearing this breed are very high because they are reared in an intensive system in which they are housed throughout their life and fed with high-quality roughage and concentrate. Therefore, the calf value of this breed is approximately four times that of a Holstein–Friesian in Japan. The beef production system in Japan, as well as other countries, is mainly divided into two operations: the cow–calf operation and feedlot operation. Recently, the number of breeding farms has decreased, and the size of each farm has increased [3], and to decrease the burden on small farmers in cow–calf operations and improve the growth efficiency of calves, a new type of operation was introduced called backgrounding. In this operation, calves aged 3–4 months are introduced and raised until they are aged 10 months. Calves that come from various cow–calf operations are reared in an intensive freestall system that contains five to seven calves in each stall, and there is the possibility of the calves being infected with diseases such as bovine respiratory disease (BRD) after their introduction. BRD is the most common and costly disease in beef cattle worldwide [4,5], and our previous studies determined that BRD incidence in backgrounding was 54.6% [6] and that a single calf with BRD incurred an annual cost of JPY 10,000 (yen) [7]. To prevent BRD, it is important to remove risk factors associated with BRD incidence and to develop an early detection system to find calves with any problems at an early stage.

Recently, many wearable sensor devices have been developed to detect physiological and behavioral changes. Our research team has developed a wearable wireless sensor to monitor the ventral tail base surface temperature (ST). This approach is potentially less invasive and stressful to the animals, and the wearable wireless ST sensor can be used for calving prediction [8,9,10] and estrus detection [11] by monitoring a substantial change in ST. However, the use of this technology is limited to adult cows, and no study has been conducted in calves. An increased ST can be used as an indicator of calves that had problems such as BRD. In previous studies, researchers reported that the circadian rhythm influenced ST [9,11]. Thus, the objective is to investigate an alternative indicator for the actual rectal temperature by examining the ventral tail base ST and assessing the early detection system of Japanese Black calves with fever using machine learning algorithms.

## 2. Materials and Methods

The present study was conducted on a backgrounding farm in Miyazaki, Japan, which is located on the southeastern coast of Kyushu and is a major cow–calf producing region. The farm collects Japanese Black calves aged 3–4 months from cow–calf operations and raises them until they are aged 10 months. The farm receives 40–50 calves from cow–calf operations every month. The calves are assigned separately to freestalls (4.7 m × 8.9 m) based on their sex and arrival weight, with each freestall containing 5–7 calves. All animal procedures in this study were approved by the Animal Care and Use Committee of the University of Miyazaki (#2020–043).

This study conducted two experiments (experiments 1 and 2). Experiment 1 was conducted to investigate an alternative indicator for the actual rectal temperature by examining the associations of the actual rectal temperature with four ST indicators because, in previous studies, researchers reported that the circadian rhythm influenced ST [9,11]. Experiment 2 was conducted to detect calves with fever, using machine learning algorithms as best indicator of temperature.

**Figure 1:**
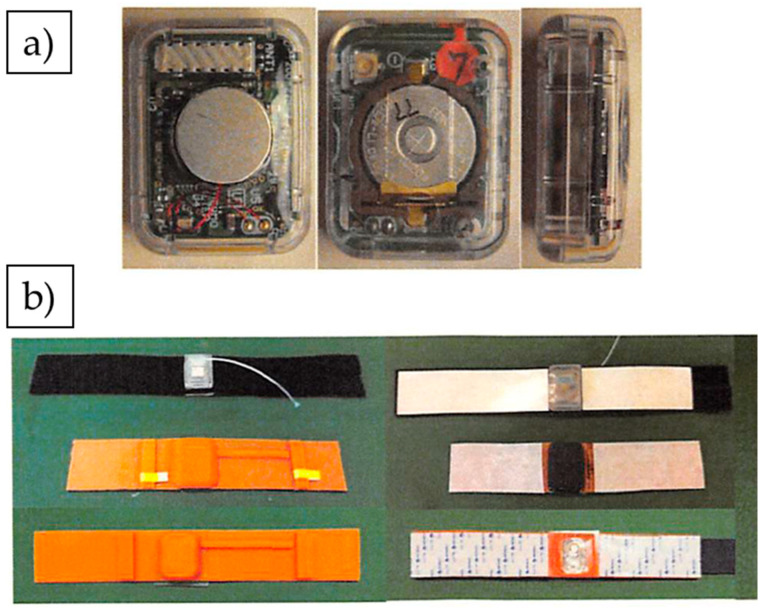
(**a**) Wearable wireless ST sensor; and (**b**) materials that attached the sensor to the tail surface.

**Figure 2:**
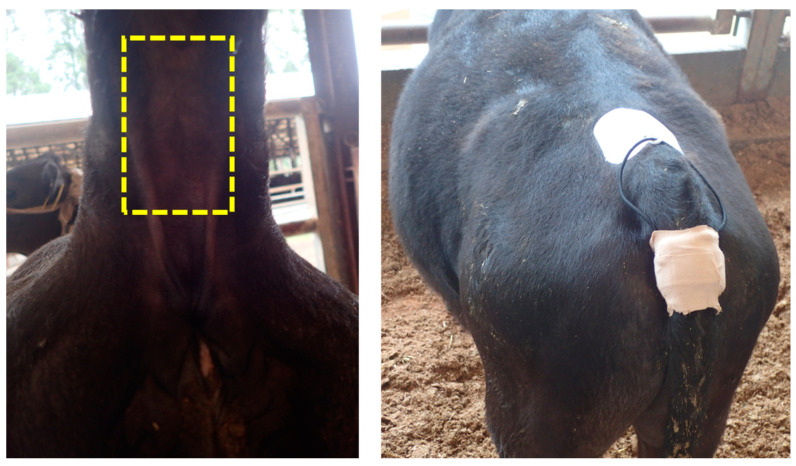
Sensor attachment area. Yellow square in the picture on the left indicates the sensor attachment area and the picture on the right shows the sensor unit wrapped with elastic medical bandages.

In experiment 1, 34 calves aged 3–4 months were used. The wearable wireless tail ST sensor was attached to the surface of the ventral tail base of each calf at its introduction to the farm (Figure 1). The sensor was attached according to the description in previous studies [9,12,13]. Briefly, the ST sensor (20.0 mm × 26.0 mm × 10.0 mm, weighing 5.5 g) was covered with urethane gel to prevent skin damage. Then, the silicone rubber belt with the ST sensor was attached to the ventral tail base. To stabilize the position of the ST sensor, an outer belt with an adjustable hook-and-loop fastener strap was surrounded and wrapped with elastic medical bandages (SRPH75 and SRPH50, Nichiban, Tokyo, Japan) (Figure 2). The ST was measured every 10 min for seven days. In experiment 2, 119 calves aged 3–4 months were used, and the wearable wireless tail ST sensor was attached in the same manner described for experiment 1. The ST was measured every 10 min for one month.

In experiment 1, the actual rectal temperature was measured twice a day (around 8–9 h and 16–17 h) by farm staff during the experimental period. To investigate the associations of the actual rectal temperature with ST, this study used four measurements: (1) raw ST values, (2) estimated ST (3) residual ST (rST), and (4) estimated rST. The estimated ST was calculated using the following equation obtained by experimental infection of Holstein–Friesian calves with BRD [14]:

Estimated ST = Tem + (Max_Tem24h 󒈒 Tem) × ((Max_Tem24h 󒈒 Min_Tem24h 󒈒 0.9)/(Max_Tem24h 󒈒 Min_Tem24h), where Tem denotes the raw ST values, Max_Tem24h denotes the maximum raw numbers of ST for the past 24 h period, and Min_Tem24h denotes the minimum raw numbers of ST for the past 24 h period. The rST was calculated as the ST minus the mean ST for the same time on the previous 3 days. The estimated rST was combined with the estimated ST and rST calculated using the estimated ST minus the mean estimated ST for the same time on the previous 3 days. A general linear model with a repeated measure (PROC GLM, SAS version 9.4; SAS Institute, Cary, NC, USA) was used to test the association between the actual rectal temperature and each measurement. The four measurements used in experiment 1 were the values closest to the time in which the rectal temperature was measured.

In experiment 2, the estimated rST was used to detect calves with fever. Data preprocessing, which is a data mining process that transforms raw data into a useful format, was performed to use the ST data for analysis. Missing or extreme values, such as ≤30 °C, ≥45 °C, and ±1 °C change in consecutive records and were excluded. Then, the maximum hourly ST was extracted to minimize the effects of a rapid change in ST in the raw data. Fever was defined as an increase of ≥1.0 °C for the estimated rST of a calf for 4 consecutive hours. To explore the association between fever and the rST variables, supervised machine learning algorithms that use a random forest were used. The original data were randomly and uniformly divided into a training (80%) and independent test set (20%); the data were then implemented by an internal repeated 10-fold cross-validation process to estimate model performance to prevent overfitting and the artificial inflation of accuracy. In this model, 20 features that consisted of the minimum and maximum value of ST during the last 3, 6, 12, 24, and 48 h, and the difference between the current minimum or maximum ST and past minimum or maximum ST during the last 3, 6, 12, 24, and 48 h, were used. Additionally, information about the calf at its introduction to the backgrounding farm, such as sex, season at introduction, arrival age, arrival weight, chest circumference at arrival, and blood line, were examined. The prediction of a calf having a fever every hour was estimated. The threshold that classified a calf as a case was a probability of more than 50%. The number of trees for the forest was set as 100. Accuracy, precision, and sensitivity were calculated as (true positives + true negatives)/(true positives + true negatives + false positives + false negatives), true positives/(true positives + false positives), and true positives/(true positives + false negatives), respectively.

## 3. Results

**Figure 3:**
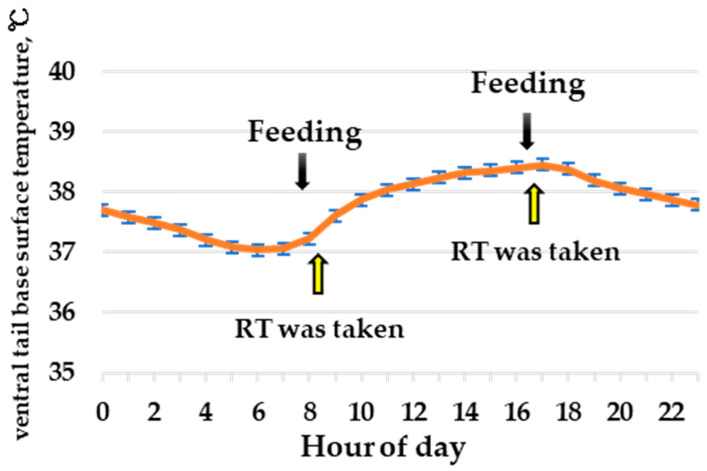
Temporal change in the mean ventral tail base surface temperature. Black arrows indicate the time of feeding, whereas yellow arrows indicate the time in which rectal temperature (RT) was taken.

**Figure 4:**
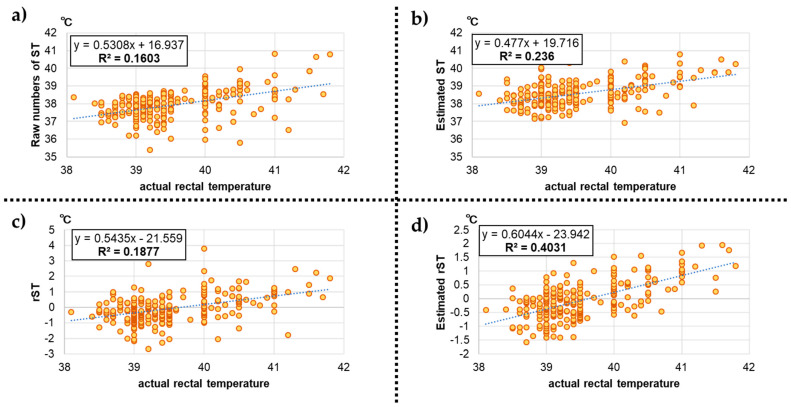
Associations of actual rectal temperatures with the four measurements: (**a**) raw surface temperature (ST) values; (**b**) estimated ST; (**c**) residual ST (rST); and (**d**) estimated rST.

In experiment 1, a diurnal variation in ST was observed (Figure 3). The lowest ST was observed at approximately 6 am, which was morning feeding time, and the highest was at approximately 5 pm, which was evening feeding time. Figure 4 shows the associations of the actual rectal temperatures with the four measurements. Regarding the raw numbers of ST, estimated ST, and rST, the R-squared values were relatively low, that is, 0.16, 0.23, and 0.19, respectively, whereas the r-squared between the actual rectal temperature and estimated rST was 0.40, which indicates that the estimated rST can be used as an indicator of the actual rectal temperature.

**Figure 5:**
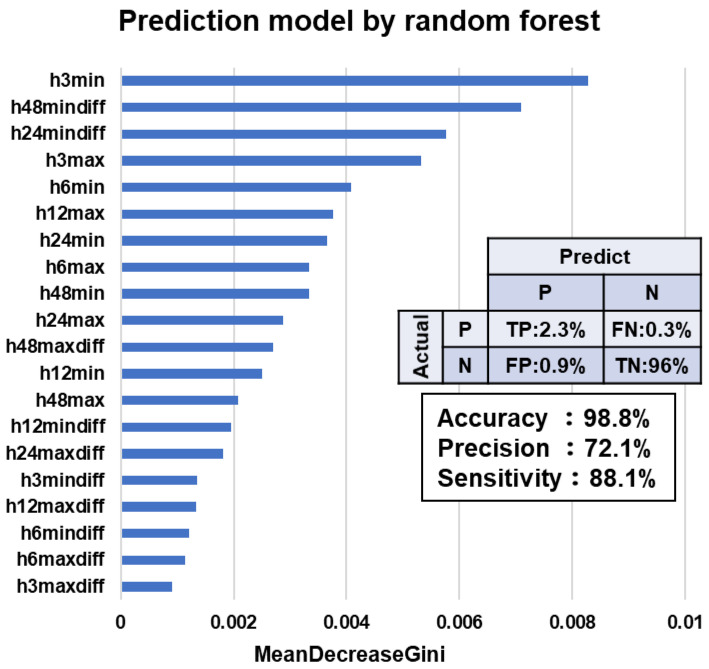
Variable importance obtained using a random forest. Variables are ordered by their importance as estimated by the random forest model. P indicates positive and N indicates negative. The terms h3min, h6min, h12min, h24min, and h48min denote the minimum value of surface temperature (ST) during the last 3, 6, 12, 24, and 48 h, respectively. The terms h3max, h6max, h12max, h24max, and h48max denote the maximum value of ST during the last 3, 6, 12, 24, and 48 h, respectively. The terms h3mindiff, h6mindiff, h12mindiff, h24mindiff, and h48mindiff denote the difference between the current minimum ST and past minimum ST during the last 3, 6, 12, 24, and 48 h, respectively. The terms h3maxdiff, h6maxdiff, h12maxdiff, h24maxdiff, and h48maxdiff denote the difference between the current maximum ST and past maximum ST during the last 3, 6, 12, 24, and 48 h, respectively.

In experiment 2, the variable importance scores (mean decrease in the Gini index) that represented the most important predictors for the detection of calves with fever were the minimum and maximum values during the last 3 h and the difference between the current value and 24- and 48-h minimum (Figure 5). For this prediction model, accuracy, precision, and sensitivity were 98.8%, 72.1%, and 88.1%, respectively. Additionally, the inclusion of information about the calf at its introduction to the backgrounding farm, such as sex, season at introduction, arrival age, arrival weight, chest circumference at arrival, and blood line, improved prediction accuracy—accuracy: 99.0%, precision: 75.2%, and sensitivity: 90.0%.

## 4. Discussion

In the present study, the ventral tail base ST was measured to detect calves with fever. Results in the present study indicate that calves with fever can be detected by monitoring the ventral tail base ST using a wearable wireless sensor. To the best of our knowledge, this is the first report on the use of a wearable wireless ST sensor attached to the ventral tail base of Japanese Black calves, although the use of this sensor was reported in cows to predict calving [8,9,10] and estrus [11] by monitoring a substantial change in the ST of cows. Many commercial wearable devices have been used for health management [15] by monitoring physiological and behavioral changes; however, most of these devices were developed for cows. The sensor used in the present study can be useful to monitor the ST of calves and detect any problems at an early stage.

The ventral tail base ST was analyzed using supervised machine learning algorithms that use a random forest to detect calves with fever. A random forest approach is one of the most precise prediction methods among machine learning approaches, which has advantages such as the ability to determine variable importance and ability to model complex confounding or interactions among independent variables [16,17]. In this study, accuracy of 98.8% and sensitivity of 88.1% indicate that the model can detect fever periods accurately. Among the variables identified in this study, the value of ST during the last 3 h and the difference between the current ST and past ST during 24 and 48 h were relevant, whereas the difference during 3–12 h was not particularly relevant. These results indicate that the use of the difference in ST during a short period is not effective for the detection of fever. 

Although a wearable wireless ST sensor can be used for the health monitoring of calves, there are several problems regarding using this technique in the field. First, this study used the estimated rST as an alternative measurement of the actual rectal temperature. This measurement had the highest consistency with the actual rectal temperature among candidate indicators; however, some calves with high actual rectal temperature had low values of estimated rST. Indeed, the R-squared values were relatively weak. Because there was a diurnal variation in raw numbers of ST, rST should be preferred over ST; however, producers or farm staff should understand that the estimated rST cannot completely reflect the actual rectal temperature. In this study there was no apparent seasonal variation, but the environmental temperature might have affected the ST. Additionally, it is important that all people related to a commercial farm, such as farm staff, farm manager, clinical veterinarian, and technical manager, should be taught how to interpret the results of analysis or alerts provided by the prediction model to avoid the misleading idea that technology cannot change animals and people; it can change the way we farm, and change animal and farmer wellbeing [18].

In the prediction model for fever, the inclusion of information about the calf at its introduction to the backgrounding farm, such as sex, season at introduction, arrival age, arrival weight, chest circumference at arrival, and blood line, improved prediction accuracy. These factors are reportedly associated with the occurrence of BRD [19,20,21,22,23,24,25,26,27,28,29]. Therefore, an ST sensor can be used on high-risk calves to detect the clinical signs of BRD at the earliest opportunity. The early detection of calves infected with BRD can reduce the use of antimicrobial agents in the field and alleviate a substantial worldwide problem from the viewpoint of antimicrobial resistance. In a previous study, researchers conducted cost–benefit analysis and found that producers with high rates of BRD can benefit financially from implementing preventative measures [27].

## 5. Conclusions

The present study indicated that an ST sensor can detect changes in an animal’s body temperature that might indicate a change in the health status of the animal. The early detection of calves with fever can be predicted by the estimated rST and past sensing data. These findings could contribute to the design and implementation of effective prevention or control of BRD, and finally decrease labor and the burden on clinical veterinarians.

## Data Availability

The data presented in this study are available upon request from the corresponding author. The data are not publicly available because of privacy issues.
